# A Multi-Cohort Examination of the Independent Contributions of Maternal Childhood Adversity and Pregnancy Stressors to the Prediction of Children’s Anxiety and Depression

**DOI:** 10.1007/s10802-022-01002-3

**Published:** 2022-12-03

**Authors:** Amanda Noroña-Zhou, Michael Coccia, Alexis Sullivan, Thomas G. O’Connor, Brent R. Collett, Karen Derefinko, Lynette M. Renner, Christine T. Loftus, Danielle Roubinov, Kecia N. Carroll, Ruby H. N. Nguyen, Catherine J. Karr, Sheela Sathyanarayana, Emily S. Barrett, W. Alex Mason, Kaja Z. LeWinn, Nicole R. Bush

**Affiliations:** 1 Department of Psychiatry and Behavioral Sciences, Weill Institute for Neurosciences, University of California, San Francisco (UCSF), San Francisco, CA USA; 2 Department of Pediatrics, UCSF, San Francisco, CA USA; 3 Center for Health and Community, UCSF, San Francisco, CA USA; 4 Departments of Psychiatry, Psychology, Neuroscience, Department of Obstetrics & Gynecology, University of Rochester, Rochester, NY USA; 5 Department of Psychiatry & Behavioral Sciences, University of Washington, Seattle Children’s Research Institute, Seattle, WA USA; 6 Department of Preventive Medicine, University of Tennessee Health Science Center, Memphis, TN USA; 7 School of Social Work, University of Minnesota, Minneapolis, MN USA; 8 Department of Occupational and Environmental Health Sciences, University of Washington, Seattle, WA USA; 9 Departments of Environmental Medicine and Public Health and Pediatrics, Icahn School of Medicine at Mount Sinai, New York, NY USA; 10 Division of Epidemiology and Community Health, University of Minnesota, Minneapolis, MN USA; 11 Department of Occupational and Environmental Health Sciences, Department of Pediatrics, University of Washington, Seattle, WA USA; 12 Department of Biostatistics and Epidemiology, Rutgers School of Public Health; Environmental and Occupational Health Sciences Institute, Rutgers University, New Brunswick, NJ USA

**Keywords:** Child anxiety, Child depression, Childhood trauma, Pregnancy stress, Intergenerational transmission

## Abstract

Women’s social experiences can have long-term implications for their offspring’s health, but little is known about the potential independent contributions of multiple periods of stress exposures over time. This study examined associations of maternal exposure to adversity in childhood and pregnancy with children’s anxiety and depression symptoms in a large, sociodemographically diverse sample. Participants were 1389 mother-child dyads (child age *M* = 8.83 years; *SD* = 0.66; 42% Black, 42% White; 6% Hispanic) in the ECHO-PATHWAYS Consortium’s three U.S. pregnancy cohorts. Women reported their exposure to childhood traumatic events (CTE) and pregnancy stressful life events (PSLE). Children self-reported on their symptoms of anxiety and depression at age 8–9 years. Regression analyses estimated associations between maternal stressors and children’s internalizing problems, adjusting for confounders, and examined child sex as a modifier. Exploratory interaction analyses examined whether geospatially-linked postnatal neighborhood quality buffered effects. In adjusted models, PSLE counts positively predicted levels of children’s anxiety and depression symptoms ([ß_Anxiety_=0.08, 95%CI [0.02, 0.13]; ß_Depression_=0.09, 95%CI [0.03, 0.14]); no significant associations were observed with CTE. Each additional PSLE increased odds of clinically significant anxiety symptoms by 9% (95%CI [0.02, 0.17]). Neither sex nor neighborhood quality moderated relations. Maternal stressors during pregnancy appear to have associations with middle childhood anxiety and depression across diverse sociodemographic contexts, whereas maternal history of childhood adversity may not. Effects appear comparable for boys and girls. Policies and programs addressing prevention of childhood internalizing symptoms may benefit from considering prenatal origins and the potential two-generation impact of pregnancy stress prevention and intervention.

**Supplementary Information:**

The online version contains supplementary material available at 10.1007/s10802-022-01002-3.

Social experiences over the life course, and particularly during sensitive periods of development, can shape one’s biology and behavior in a manner that has long-term physical and mental health implications (Davis & Narayan, 2020; Felitti et al., 1998; Hentges et al., 2019). “Sensitive periods” are developmental windows during which biological systems (e.g., central nervous system, hypothalamic-pituitary-adrenal [HPA] axis, immune system) undergo rapid growth and organization and evidence heightened malleability to environmental inputs. The fetal, childhood, and pregnancy periods are established sensitive periods over the life course (Aschbacher et al., 2021; Davis & Narayan, 2020). Experiences of stress and adversity, defined as experiences or conditions that threaten, or are perceived to threaten, physiological equilibrium (Loman & Gunnar, 2010; Weinstock, 2005), during sensitive periods of development may have especially enduring impacts on health (Davis & Narayan, 2020). Accumulating work suggests that these impacts may span generations and play an important role in the development of children’s behavioral and emotional health outcomes. Given the high prevalence of childhood adversity among adult women^1^ (Frankenberger et al., 2015; Mersky & Janczewski, 2018) and exposure to violence and stress during pregnancy (Burns et al., 2015), efforts to elucidate their long-term, intergenerational impacts would not only inform the provision of child mental health care but also the improvement of population health across the life course.

Mental health is a key component of population health. Anxiety and depression, beginning in childhood and throughout the life course, are leading causes of global health-related burden (Vos et al., 2020). The onset of anxiety and depression sharply increases during middle childhood (5–11 years of age; Kessler et al., 2012), and childhood onset of such internalizing symptoms often becomes chronic or relapsing into adulthood (Jakobsen et al., 2012; Pine et al., 1998). Thus, identification of risk and protective factors for internalizing problems during middle childhood, including factors preceding the child’s birth, is critical for *preventing* long-standing distress and impairment to individuals, as well as economic costs to society.

Recent examinations have reported that maternal childhood adversity (Rowell & Neal-Barnett, 2022) and stress during pregnancy (Clayborne et al., 2021; Hentges et al., 2019) are each associated, as separate predictors, with children’s increased risk for internalizing problems, suggesting that there may be intergenerational transmission of risk and pointing to the potential value of better understanding these associations. While a long line of literature has demonstrated prenatal origins of child mental and physical health, very few studies have simultaneously examined maternal stress exposures during *and* prior to pregnancy. To advance etiological understanding of child anxiety and depression and help pinpoint the most impactful developmental windows for upstream trauma prevention, a life course perspective that simultaneously considers maternal exposure to adversity during *multiple* sensitive periods of her own development is needed. Further, extant intergenerational work has focused primarily on *risk* factors for child anxiety and depression, with little consideration of factors that may protect or support individuals exposed to such risk. In order to develop broadly impactful programs and policies that benefit the mental health of children and their families, examinations of community-level modifiable sources of potential resilience and protection for children are needed. The current study’s aims were to advance the work in this space through examination of the unique effects of maternal stress exposure during childhood and pregnancy, well-powered tests of sex differences, and the potential protection conferred by residing in a well-resourced neighborhood.

## Intergenerational and Developmental Origins of Health and Disease

A long-studied framework for examining the impact of maternal stress exposures on offspring health is the concept of developmental origins of health and disease (DOHaD; Barker, 1998). The DOHaD framework posits that maternal experiences of stress carry long-term implications for her child’s health, in part through maternal-placental-fetal biological pathways (Entringer et al., 2012; O’Brien et al., 1999; Van den Bergh et al., 2020). Due to the rapid pace of fetal development, fetuses are particularly sensitive to intrauterine inputs (such as maternal circulating stress hormones and immune activity) and adapt with changing inputs in order to maximize survival outside of the womb (Entringer et al., 2015) in ways that have lifelong implications for their development and health (Noroña et al., 2020). While the preponderance of initial DOHaD research demonstrated offspring impacts secondary to maternal exposure to stress during pregnancy, more recently, research has shown that this intergenerational cascade of stress effects may extend back to maternal childhood adversity as well (Gray et al., 2017). Consideration of significant life stressors prior to conception is an important direction of DOHaD science, as sole focus on prenatal exposures may obscure the impact of earlier risk exposures on offspring health. Related work has shown that childhood adversity exposure has lasting effects on the functioning of women’s biological systems during adulthood (Aschbacher et al., 2021), and pregnancy specifically (Steine et al., 2020), in ways that have direct relevance for fetal development. Such evidence provides additional support for the need to simultaneously consider maternal adversity exposure during and prior to pregnancy to understand intergenerational transmission of stress effects.

## Maternal Exposure to Childhood Adversity and Offspring Internalizing Problems

More than half of adult women in the United States report exposure to childhood adversity, which includes physical, sexual, and emotional abuse, neglect, and household dysfunction (Frankenberger et al., 2015; Mersky & Janczewski, 2018). A recent systematic review identified twelve studies of maternal childhood adversity and offspring internalizing problems, and findings across these studies consistently showed positive associations between maternal childhood adversity and their child’s internalizing problems (Rowell & Neal-Barnett, 2022). Across the twelve studies, childhood internalizing was assessed entirely via parent report, and child ages ranged from early childhood to adolescence. Though this body of work is promising, Rowell and Neal-Barnett (2022) highlighted underrepresentation of low-income families and families of color in the extant evidence base limits their generalizability. Studies in this growing area have predominantly leveraged either large, mostly White, low-risk epidemiological samples from outside of the U.S. or small (*N* < 70), sociodemographically diverse American samples. A large, sociodemographically and geographically diverse epidemiological sample would enhance generalizability of these findings to the social and policy context of the U.S. and afford the development of equitable solutions for child mental health.

## Maternal Exposure to Stress During Pregnancy and Offspring Internalizing Problems

Women’s rate of exposure to major adversities during pregnancy is unfortunately high, as nearly three-quarters of mothers report at least one major life stressor, such as separation from their partner, death of a loved one, and job loss, in the 12 months before childbirth (Burns et al., 2015). Major stress exposures can directly impact maternal mental and physical health during pregnancy (Dayan et al., 2010; László et al., 2013) and indirectly impact a range of offspring outcomes in childhood, including externalizing problems, internalizing problems, and physical illness (Bush et al., 2020; Van den Bergh et al., 2020). In closer examination of links with childhood internalizing problems, maternal exposure to stress during pregnancy, ranging from perceived psychosocial distress to severe exogenous environmental events such as floods, has been associated with increased risk of offspring anxiety and depression (Clayborne et al., 2021; Hentges et al., 2019; McLean et al., 2018). Though this body of work is compelling, with some exceptions, most studies have focused on outcomes in young children and relied on parent reports, raising concerns about time-limited programming effects and potential rater bias in tested associations (De Los Reyes et al., 2015). In addition, despite evidence that maternal exposure to major life events during pregnancy is associated with child outcomes, even after adjusting for maternal subjective ratings of psychological distress during pregnancy (Rudd et al., 2022), most studies have relied on maternal self-report of subjective distress rather than more objective accounts of stressor exposures.

To date, only two studies have simultaneously examined maternal childhood adversity and constructs related to pregnancy stress in the prediction of childhood internalizing symptoms (Letourneau et al., 2019; Thomas-Argyriou et al., 2021). Measurement of maternal pregnancy stress exposure varied; one study used a biomarker of HPA axis functioning during pregnancy (Thomas-Argyriou et al., 2021) and the other assessed maternal mental health (anxiety and depression) during the perinatal period (Letourneau et al., 2019). Both studies found that maternal exposure to childhood adversity and pregnancy/perinatal stress-correlates each independently predicted parent-reported offspring internalizing symptoms, providing compelling foundational support for these relations. However, neither of these prior works examined child internalizing problems beyond the preschool period, which precedes the typical developmental timing of onset of internalizing symptoms. Thus, investigation into the longevity of observed associations is merited, particularly with child self-reported symptoms. The current study addresses these gaps through testing associations between maternal exposures to childhood and pregnancy stressors and child-reported anxiety and depression in middle childhood.

## Variability in Associations Between Maternal Stress Exposure and Child Internalizing

Not all families or individuals exposed to adversity suffer negative outcomes (Baldwin et al., 2021). Ecological systems theory emphasizes that individual-level factors (e.g., sex, age, temperament) and nested, interconnected environmental contexts (e.g., family, school, neighborhood) interact with social experiences to impact child development over time (Bronfenbrenner, 1994). Thus, when possible, tests of stress effects on children should consider such individual- and context-level influences. Child biological sex and neighborhood quality are considered in the current study.

Child biological sex may affect the impact of maternal stress exposures on child mental health, with evidence pointing to the intrauterine hormonal milieu as guiding the development of such potential sex differences (Bale & Epperson, 2015). Despite numerous theoretical perspectives positing sex differences and non-human animal literature supporting it, empirical examinations of maternal exposure to stressors during childhood or pregnancy and sex differences in offspring internalizing have been inconclusive. While some prior work has found evidence for differential effects by sex (Davis & Pfaff, 2014; Letourneau et al., 2019), others have not (O’Donnell et al., 2014), highlighting the need for additional inquiry, particularly in adequately powered, diverse samples. Sex differences are also relevant to the development of internalizing disorders. Depression is more common among girls than boys in adolescence (Altemus et al., 2014), and it is possible that these effects have intergenerational and/or prenatal roots. Thus, examination of contributions of maternal stress exposures in interaction with sex as children age into the middle childhood period may shed light on the development of sex differences in child depression and anxiety.

Moving to environment-level influences, previous work considering the social ecological contexts that influence how maternal stress affects child development has mostly focused on promotive or buffering factors in the family, such as parent mental health and parenting (Ahmad et al., 2022; Eckshtain et al., 2019; Grant et al., 2010). While valuable for informing family-based psychosocial interventions, family factors are more difficult to rigorously assess, such as through direct observation or clinical interviewing, in large cohort studies. It is important to increase focus on the role of broader ecological systems that impact caregiving and expand the burden of responsibility for health promotion beyond individual caregivers to include larger structural programs and policies with wide-ranging impacts on all families. Examinations of community-level protective factors, such as neighborhood quality, have the potential to inform universal health promotion and policy.

Adverse neighborhood attributes (e.g., poor physical maintenance, crime) have been linked to greater anxiety and depression in children (Butler et al., 2012) and stress and depression in pregnant women (Giurgescu et al., 2015), findings that highlight the role of neighborhoods for internalizing problems in families. A few health-promotion-oriented studies have found that positive neighborhood attributes (e.g., social cohesion) may buffer associations between postnatal family-level adversity (e.g., maternal depression, harsh parenting) and child mental health (Delany-Brumsey et al., 2014; Silk et al., 2004), but no previous work has examined whether neighborhood quality buffers the effects of maternal stress exposure prior to the child’s birth on offspring psychopathology.

The current study sought to advance the literature on the development of childhood anxiety and depression through an approach that combines intergenerational, life course, and ecological systems frameworks. We address a range of current gaps in the literature through simultaneous consideration of maternal history of exposure to major stressors in both her childhood and pregnancy, use of a large sociodemographically and geographically diverse sample, child self-report of their internalizing symptoms, consideration of sex-specific associations, and examination of an objective neighborhood-level protective factor. Primary aims were to: (a) examine unique associations between maternal exposure to adversities during childhood and pregnancy and offspring’s anxiety and depression symptoms at age 8–9, and (b) determine whether these associations differed by child biological sex. Given prior findings that maternal childhood adversity and pregnancy stressors each predict mental health problems in childhood, we expected both factors would independently predict both outcomes. As the evidence for sex moderation has been inconsistent, we did not hypothesize a direction for those potential interactive effects. In addition, in the first exploration of neighborhood quality as a potential buffer of intergenerational stress effects on children’s mental health, we examined a geospatially-linked assessment of neighborhood-level resources, as a moderator of the relations between maternal adversity and child internalizing. We pursued these aims by utilizing harmonized data from the ECHO-PATHWAYS Consortium (https://deohs.washington.edu/echo), a large, multi-cohort longitudinal sample of sociodemographically diverse dyads across six U.S. geographic regions.

## Method

### Participants

Participants were 1389 mother-child dyads with outcome data who were enrolled in one of three prospective pregnancy cohorts participating in the U.S.-based NIH ECHO-PATHWAYS Consortium: TIDES, CANDLE, and GAPPS (LeWinn et al., 2022).

### Procedures

The TIDES study recruited women 18 years or older with a healthy pregnancy from four university-based prenatal clinics: the University of San Francisco, California (San Francisco, CA), the University of Rochester Medical Center (Rochester, NY), the University of Minnesota (Minneapolis, MN), and the University of Washington/Seattle Children’s (Seattle, WA; Barrett et al., 2014). In the CANDLE study, based at the University of Tennessee Health Science Center (Memphis, TN), healthy women between 16 and 40 years of age with low medical risk pregnancies and plans to deliver at a participating study hospital were enrolled in their second trimester of pregnancy using community-based and clinic-based recruitment (LeWinn et al., 2020). The GAPPS study enrolled pregnant women over the age of 18, attending select prenatal clinics in the Seattle and Yakima, WA areas (www.gapps.org). All three of these cohorts included research visits during pregnancy, a birth exam and/or birth record review, and postnatal visits around child age 4–6 and 8–9 years. Extant and prospectively collected individual-level data were pooled and harmonized across cohorts upon enrollment into ECHO-PATHWAYS. Mothers provided informed consent for themselves and their children. All ECHO-PATHWAYS research activities were approved by the University of Washington IRB and relevant partner institutions.

All ECHO-PATHWAYS cohorts administered measures of internalizing symptoms to children at the age 8–9 year visit. Prior to the administration, parents had an opportunity to review the survey content. Examiners read instructions and test items out loud to the child, and the child selected their response.

### Measures

**Child anxiety.** Children self-reported on their symptoms of anxiety at the age 8–9 visit using the Screen for Child Anxiety Related Emotional Disorders (SCARED; Birmaher et al., 1999), a 41-item instrument for children ages 8–18 that screens for anxiety disorders across five domains: generalized anxiety, separation anxiety, social anxiety, panic/somatic symptoms, and school avoidance. Children are asked to rate items on a scale of ‘not true or hardly ever true’, ‘somewhat true or sometimes true’, and ‘very true or often true’ that describe them best for the last 3 months. Items are scored from 0 to 2 and summed to create an overall total score, with higher scores indicating greater anxiety symptoms. The SCARED has good internal consistency, test-retest reliability, and discriminant validity (Birmaher et al., 1999); in the current sample, SCARED internal consistency was good (α=0.91). Scores were analyzed continuously (range = 0–82) and dichotomously, using the established cut off score of 30 to indicate the presence of clinically significant anxiety. SCARED protocols missing over 10% of items were considered invalid and excluded from analyses. A prorated total score was generated when ≤ 4 items were missing.

**Child depression.** Children self-reported on depression symptoms at the age 8–9 visit using the Children’s Depression Inventory, Second Edition (CDI-2; Kovacs, 2011), a 28-item instrument for children ages 7–17 that assesses cognitive, affective, and behavioral signs of depression within the past two weeks (e.g., feelings of worthlessness, anhedonia, and suicide ideation). Items are scored from 0 to 2 and summed to create an overall total score, with higher scores indicating greater depression symptoms. The CDI-2 has good internal consistency, test-retest reliability, and discriminant validity (Kovacs, 2011); in the current sample, CDI-2 internal consistency was good (α=0.83). Scores were analyzed continuously (range = 0–56) and dichotomously, using the clinical cut-off scores of 14 for girls and 17 for boys in accordance with manual guidelines. CDI-2 protocols missing over 10% of items are considered invalid and were excluded from analyses. Total scores were prorated according to the CDI-2 manual guidelines in cases where ≤ 3 items were missing.

**Maternal childhood trauma exposure (CTE).** Exposure to traumatic adversities involving sexual or physical violence during childhood was assessed via three available items from the Traumatic Life Events Questionnaire (Kubany & Haynes, 2000) that assess events during childhood. During a postnatal follow-up assessment (age 4–6 for GAPPS, age 6 for TIDES, age 6 for CANDLE), women retrospectively reported whether they were physically abused before age 18 years, witnessed family violence before age 18 years, or experienced sexual abuse before age 13 years. Scores were calculated for participants who completed the entire survey; affirmative responses were summed into a CTE count variable (range = 0–3). This three-item measure of CTE has been used in prior work and been shown to be associated with a range of maternal-child outcomes (Ahmad et al., 2021; Browne et al., 2022; Roubinov et al., 2022; Slopen et al., 2018).

**Pregnancy stressful life events (PSLE).** During a postnatal follow-up assessment (age 4–6 for GAPPS, age 6 for TIDES, age 8 for CANDLE), women reported retrospectively on the number of types of stressful life events they experienced during pregnancy using a list of 14 events adapted from the Centers for Disease Control and Prevention Pregnancy Risk Assessment Monitoring System (PRAMS) survey (Whitehead et al., 2003). See Supplement for a list of the 14 PSLE items. Scores were calculated for participants who completed the entire survey; affirmative responses were summed into a PSLE count variable (range = 0–14). Evaluations of retrospective reports of major life events have established their validity and robustness to bias (Ramos et al., 2020; Reuben et al., 2016).

**Neighborhood quality.** ​​The Childhood Opportunity Index (COI) is a publicly available tool that evaluates multi-domain neighborhood-level risks and resources for large U.S. metropolitan areas (Acevedo-Garcia et al., 2020; Noelke et al., 2020). Neighborhoods are identified by census tract and given an overall “opportunity” score based on 29 indicators that span three domains relevant to children’s health and development: educational opportunity, health and environmental opportunity, and social and economic opportunity. Educational opportunity is reflected by 11 indicators that include school poverty rate, student math and reading proficiency levels, and proximity to licensed early childhood education centers. Health and environmental opportunity is reflected by 10 indicators, including proximity to toxic waste sites, proximity to parks, and availability of healthy food retailers. Social and economic opportunity is reflected by 8 indicators that include foreclosure rate, poverty rate, and proximity to employment. Each of the indicators was constructed using data collected from large-scale, nationally representative surveys (e.g., U.S. Census Bureau American Community Survey, U.S. Environmental Protection Agency Toxic Release Inventory) and converted into a standardized score (z-score) due to their varying units of measurement. The COI developers derived weights for each of the indicators based on the magnitude of their associations with child health outcomes. Consistent with prior work on the effects of neighborhood poverty on child health (Duncan & Menestrel, 2019), social and economic factors (e.g., neighborhood rates of poverty, employment, and homeownership) carry the most weight in the overall COI score. Complete information about the raw data sources and the methods for indicator derivation is available at http://www.diversitydatakids.org.

**Covariates.** The ECHO-PATHWAYS cohorts are well-characterized, allowing for robust adjustment of potential confounders and consideration of mechanistic variables on the path between maternal stress exposures and child internalizing problems. Confounding and possible mechanistic variables were determined a priori and included: study site, family income adjusted for household size and region; maternal parity, pre-pregnancy BMI, education, age, prenatal smoking, and postnatal depression; parent-report of child race and ethnicity^2^, biological sex assigned at birth, gestational age at birth, birth weight, breastfeeding history, and child age at outcome. See Supplemental text for additional details on measurement, selection, and analysis of covariates.

### Data Analytic Plan

Primary analyses examined the associations between maternal stress exposures (CTE and PSLE) and child-reported anxiety and depression symptoms in the multi-cohort sample using multiple linear regression. Based on the literature, we developed a staged adjustment approach, allowing exploration of the influence of increasing levels of adjustment on results. First, correlations were used to examine unadjusted, bivariate associations between primary predictors and outcomes (i.e., maternal CTE, PSLE, child anxiety, child depression). Next, associations were examined within minimally-adjusted models (Model 1), controlling for data-collection site, child age at outcome assessments, and child biological sex. In our primary model (Model 2), which we defined as the fully-adjusted model, a set of variables identified as potential confounders (Lund et al., 2018; Tien et al., 2020) were included as covariates: log-transformed family income adjusted-for-household-size-and-region (assessed at outcome timepoint), household size, and an interaction between income and household size; maternal parity, pre-pregnancy BMI, education, and age at enrollment; and child race and ethnicity. All non-categorical covariates were standardized (*M* = 0, *SD* = 1).

Moderation was tested in two stages: (1) child biological sex was examined using cross-product interaction terms between each stressor and sex, entered together, in fully-adjusted models (Model 2); (2) neighborhood quality (COI) was examined along with biological sex, including all two-way and a three-way cross-product terms for each stressor. As an additional consideration, we transformed continuous symptom scores into clinical threshold indicators for depression and anxiety and re-fit Model 2 using logistic regression.

In secondary analyses, we examined two extended models based on Model 2. To further specify the effects of maternal stress exposures on child internalizing, Model 2a included additional covariates that may be on the mechanistic path between stress exposures and child internalizing (Johnson & Marlow, 2011; Tien et al., 2020), including: prenatal smoking, child gestational age at birth, child birth weight, breastfeeding history, and maternal self-report of depression at child age 8–9. Finally, to address known multicollinearity between race and ethnicity and the COI (Acevedo-Garcia, 2021), Model 2b was fit without the inclusion of a race/ethnicity covariate.

The MICE (Multivariate Imputation by Chained Equations) package in R was used to impute missing values for all predictors and covariates across the three-cohort sample (see Table 1) according to methods appropriate for variable type (i.e., predictive mean matching, logistic regression, polytomous regression). All derived variables, including log-transformed household income, internalizing symptom clinical thresholds, and moderation cross-product terms, were calculated from their source variables during the imputation and passively imputed. The imputation model used all other variables as predictors for each incomplete data variable, including sex and neighborhood opportunity interaction terms. The imputed dataset was used in all primary models; thus, each model had a sample size of 1389. Finally, sensitivity analyses compared exposure effect size from primary Model 2 using the imputed dataset with a complete-cases analysis (CCA) for the pooled sample; and then a leave-one-out study recruitment site analysis.

**Table 1:** Characteristics of the analytic sample

Variable	Overall, N = 1,389^1^	CANDLE, N = 824	GAPPS, N = 119	TIDES, N = 446
**Cohort birth year, n (%)**				
2007	48 (3.5)	48 (5.8)	0 (0)	0 (0)
2008	147 (11)	147 (18)	0 (0)	0 (0)
2009	198 (14)	198 (24)	0 (0)	0 (0)
2010	218 (16)	218 (26)	0 (0)	0 (0)
2011	390 (28)	213 (26)	23 (19)	154 (35)
2012	308 (22)	0 (0)	51 (43)	257 (58)
2013	80 (5.8)	0 (0)	45 (38)	35 (7.8)
**Child age at outcome**				
Mean (SD)	8.83 (0.66)	8.87 (0.74)	8.50 (0.56)	8.85 (0.49)
Median [IQR]	8.75 [8.24, 9.25]	8.80 [8.16, 9.33]	8.36 [8.05, 8.84]	8.79 [8.44, 9.20]
Range	7.47, 10.98	8.00, 10.98	7.47, 10.37	8.02, 10.28
**Child biological sex, n (%) male**	683 (49)	407 (49)	67 (56)	209 (47)
**Ethnicity, n (%)**				
Non-Hispanic	1,283 (94)	797 (97)	101 (89)	385 (90)
Hispanic	82 (6.0)	26 (3.2)	13 (11)	43 (10)
Missing	24	1	5	18
**Race, n (%)**				
White	608 (45)	235 (29)	88 (78)	285 (66)
Black/African American	594 (44)	535 (65)	2 (1.8)	57 (13)
Asian	31 (2.3)	5 (0.6)	2 (1.8)	24 (5.6)
American Indian/Alaska Native	6 (0.4)	1 (0.1)	0 (0)	5 (1.2)
Other	97 (7.1)	39 (4.7)	19 (17)	39 (9.1)
Multiple Race	29 (2.1)	8 (1.0)	2 (1.8)	19 (4.4)
Missing	24	1	6	17
**Maternal age (years)**				
Mean (SD)	28.5 (6.1)	26.6 (5.6)	32.2 (5.8)	31.1 (5.6)
Median [IQR]	29.0 [24.0, 33.0]	26.0 [22.0, 31.0]	32.0 [29.0, 36.0]	32.0 [28.0, 35.0]
Range	16.0, 49.0	16.0, 40.0	18.0, 49.0	18.0, 44.0
Missing	7	5	2	0
**Maternal education, n (%)**				
Less than high school	53 (4.0)	37 (4.7)	1 (0.9)	15 (3.5)
High school completion	343 (26)	309 (39)	10 (8.7)	24 (5.7)
Vocational/technical school or associates degree	190 (14)	96 (12)	25 (22)	69 (16)
College degree	369 (28)	200 (25)	42 (37)	127 (30)
Graduate or professional degree	372 (28)	146 (19)	37 (32)	189 (45)
Missing	62	36	4	22
**Household income ($, annual)**				
Mean (SD)	67,133 (53,085)	41,275 (29,601)	103,604 (50,789)	103,956 (59,059)
Median [IQR]	54,990 [23,863, 85,984]	31,917 [13,831, 74,235]	101,749 [68,786, 142,449]	112,439 [55,751, 155,382]
Range	2,651, 218,749	2,651, 86,768	6,105, 188,638	5,973, 218,749
Missing	101	68	0	33
**Household count, n (%)**				
2	57 (4.4)	40 (5.1)	2 (1.7)	15 (3.6)
3	224 (17)	127 (16)	13 (11)	84 (20)
4	533 (41)	279 (36)	58 (50)	196 (48)
5	291 (22)	186 (24)	26 (23)	79 (19)
6+	204 (16)	150 (19)	16 (14)	38 (9.2)
Missing	80	42	4	34
**Birth order**				
Mean (SD)	2.51 (1.66)	2.58 (1.62)	2.68 (2.05)	2.35 (1.61)
Median [IQR]	2.00 [1.00, 3.00]	2.00 [1.00, 3.00]	2.00 [1.00, 3.00]	2.00 [1.00, 3.00]
Range	0.00, 13.00	1.00, 9.00	0.00, 13.00	1.00, 12.00
Missing	18	0	2	16
**Maternal BMI (kg/m2)**				
Mean (SD)	27 (7)	28 (8)	27 (7)	26 (7)
Median [IQR]	25 [22, 31]	26 [23, 32]	24 [22, 29]	24 [21, 29]
Range	15, 72	15, 72	18, 50	16, 59
Missing	19	1	6	12
**Child birthweight (gm)**				
Mean (SD)	3,247 (619)	3,237 (550)	2,977 (993)	3,337 (589)
Median [IQR]	3,280 [2,960, 3,625]	3,252 [2,964, 3,570]	3,197 [2,453, 3,697]	3,318 [3,030, 3,685]
Range	550, 5,154	757, 4,690	628, 4,650	550, 5,154
Missing	13	6	1	6
**Child gestational age at birth (days)**				
Mean (SD)	272 (16)	272 (13)	259 (29)	275 (14)
Median [IQR]	275 [269, 281]	274 [269, 279]	272 [245, 279]	277 [270, 284]
Range	175, 297	181, 293	176, 289	175, 297
Missing	10	5	0	5
**Breastfeeding history, n (%) any**	1,053 (77)	558 (68)	105 (91)	390 (92)
Missing	29	1	4	24
**Pregnancy smoking, n (%) any**	125 (9.0)	94 (11)	6 (5.0)	25 (5.6)
**Maternal depression symptoms (t-score)**				
Mean (SD)	46 (8)	44 (7)	48 (8)	49 (8)
Median [IQR]	45 [39, 52]	39 [39, 48]	48 [43, 53]	48 [43, 55]
Range	37, 81	39, 74	37, 74	37, 81
Missing	188	169	9	10
**Neighborhood quality (COI)**				
Mean (SD)	-0.01 (0.04)	-0.02 (0.04)	0.02 (0.02)	0.02 (0.04)
Median [IQR]	0.00 [-0.04, 0.03]	-0.03 [-0.05, 0.01]	0.02 [0.01, 0.04]	0.03 [0.01, 0.04]
Range	-0.09, 0.08	-0.09, 0.05	-0.04, 0.06	-0.09, 0.08
Missing	100	44	19	37
**Study site, n (%)**				
Memphis, TN	824 (59)	824 (100)	0 (0)	0 (0)
San Francisco, CA	115 (8.3)	0 (0)	0 (0)	115 (26)
Minneapolis, MN	120 (8.6)	0 (0)	0 (0)	120 (27)
Rochester, NY	119 (8.6)	0 (0)	0 (0)	119 (27)
Seattle, WA (TIDES)	92 (6.6)	0 (0)	0 (0)	92 (21)
Seattle, WA (GAPPS)	86 (6.2)	0 (0)	86 (72)	0 (0)
Yakima, WA	33 (2.4)	0 (0)	33 (28)	0 (0)

^1^n (%); c(“N”, “Mean (SD)”, “Median [IQR]”, “Range”)

## Results

**Descriptives.** Table 1 provides the demographic and psychosocial characteristics of the current sample. Briefly, children (51% female) were 8–9 years old (*M* = 8.83 years; *SD* = 0.66) at the time of the assessment and represented diverse racial and ethnic backgrounds. Mothers, on average, self-reported relatively low levels of depression at the outcome timepoint (t-score *M* = 46; *SD* = 8). In regard to outcome measures, 37% and 15% of children were above the clinical threshold for anxiety and depression, respectively. Predictors were positively intercorrelated (*r* = .26), as were outcomes (*r* = .53). Missing data on the predictor variables was low (5% CTE, 3% PSLE), and comparison of the maternal stress exposure measures revealed that CANDLE participants reported higher levels of PSLE than TIDES and GAPPS (p < .001). GAPPS participants reported slightly lower levels of CTE than CANDLE and TIDES (p < .05). See Supplemental Tables S1-S3 for additional descriptive statistics of exposures and outcomes.

**Predictions of child anxiety and depression symptoms.** Bivariate correlations revealed that maternal CTE had a weak, positive association with child depression symptoms (*r* = .08); PSLE positively associated with child anxiety symptoms (*r* = .15) and depression symptoms (*r* = .13). Table 2 presents results from the primary regression analyses. Minimally-adjusted regression models (Model 1) indicated that maternal CTE was not significantly associated with child anxiety or depression, but maternal PSLE was positively associated with child anxiety symptoms (*β =* 0.12, 95%CI [0.07–0.17]) and depression symptoms (*β =* 0.11, 95%CI [0.06–0.17]).

**Table 2:** Associations of CTE and PSLE with anxiety and depression symptoms and effect modification by sex in the pooled sample using multiply-imputed data

	Anxiety			Depression		
	Model 1	Model 2	Model 3	Model 1	Model 2	Model 3
CTE	0.01 (-0.05, 0.06)	-0.02 (-0.08, 0.04)	0.03 (-0.11, 0.05)	0.02 (-0.03, 0.08)	-0.01 (-0.07, 0.05)	-0.02 (-0.06, 0.10)
PSLE	0.12 *** (0.07, 0.17)	0.09 ** (0.03, 0.14)	0.13 ** (0.05, 0.21)	0.11 *** (0.06, 0.17)	0.08 ** (0.03, 0.14)	0.12 ** (0.05, 0.20)
CTE x Sex			-0.02 (-0.09, 0.13)			-0.07 (-0.17, 0.04)
PLSE x Sex			-0.09 (-0.20, 0.02)			-0.08 (-0.19, 0.02)
R2	0.08	0.15	0.15	0.08	0.13	0.13

*Notes*. Standardized betas and 95% confidence intervals are displayed. CTE = maternal childhood traumatic events; PSLE = pregnancy stressful life events. Model 1 is minimally controlled for study recruitment site, child age at outcome assessment, and child biological sex. Model 2 additionally controlled for log-transformed family income adjusted for household size and region (assessed at outcome timepoint), household size, and an interaction between income and household size; maternal parity, pre-pregnancy BMI, education, and age at enrollment; and child ethnicity. Using the same covariates as Model 2, Model 3 includes cross-product terms for each exposure*sex. Child biological sex is coded 0 for female, 1 for male. *** p < .001; ** p < .01; + p < .1. Analytic sample *N* = 1389 for all models.

In fully-adjusted models (Model 2), CTE remained nonsignificant, and PSLE retained its independent positive associations with child anxiety (*β =* 0.09, 95%CI [0.03–0.14]) and depression (*β =* 0.08, 95%CI [0.03–0.14]). While statistically significant, PSLE effects were small in magnitude, and smaller than some covariate effects (i.e., race/ethnicity and household size for anxiety; pre-pregnancy BMI, household size, and site for depression). Model 2 accounted for 15% and 13% of variance in child anxiety and depression, respectively. Of note, PSLE effects on anxiety and depression passed correction for multiple testing (*p* < .025). See Tables S4-S5 for all regression model coefficients.

**Sex moderation.** Tests of sex moderation revealed that the associations between PSLE and child anxiety and depression symptoms did not differ by child sex (Table 2).

**Predictions of clinical thresholds.** Logistic regression results showed that each additional PSLE was related to a 9% (95%CI [1.02, 1.17]) increase in the odds of child-reported anxiety occurring at or above the clinically significant threshold (Table S5). In addition, this finding passed correction for multiple testing (*p* < .025). No associations with clinically significant depression symptoms were detected.

**Tests of neighborhood quality as a protective factor.** In bivariate analyses, COI was significantly negatively correlated with both child anxiety (*r* = − .28) and depression (*r* = − .22). However, when included in fully-adjusted regression models, COI did not have a significant main effect on either child anxiety or depression, nor did COI moderate maternal stress effects of either CTE *(p* ≥ .5) or PSLE (*p* ≥ .7). In sensitivity analyses in which the child race/ethnicity covariate was removed due to established high covariance between COI and race/ethnicity (see Supplemental Tables S3-S4, Model 2b), the COI interaction term remained nonsignificant for both outcomes, though the negative main effect of COI on child anxiety became significant.

**Sensitivity analyses.** We estimated the influence of specific study sites on the pooled results in leave-one-site-out sensitivity analyses (see Figures S1-S2 in Supplement). Patterns of association were mostly similar, except the PSLE effect size was larger when removing the Memphis site (the largest subsample). Sensitivity analysis using listwise-deletion (*n* = 1118 for anxiety, *n* = 1116 depression) were consistent with the results of the multiple regression using MI (*N* = 1389), as shown in Figures S1-S2.

## Discussion

An intergenerational, developmental lens is critical to understand the etiology of and best prevention and intervention strategies for children’s anxiety and depression. In a large, sociodemographically diverse, multi-cohort, multi-site, longitudinal sample of mother-child dyads, the current study examined whether maternal exposure to adversity during her own childhood and pregnancy predicted children’s internalizing problems at age 8–9. When examined simultaneously, maternal exposure to pregnancy stressful life events (PSLE), but not maternal history of childhood traumatic events (CTE), uniquely predicted children’s self-reported anxiety and depression symptoms, as well as their likelihood of clinically significant anxiety, after adjustment for a range of confounders and possible mediators. Neither child biological sex nor objectively-assessed neighborhood quality moderated the effects of maternal stress on child internalizing symptoms. These findings suggest that maternal history of childhood trauma may not have a measurable, sustained association with children’s internalizing symptoms at age 8–9, whereas maternal exposure to stressors during pregnancy does predict child anxiety and depression at this age. Further, these effects appear comparable across boys and girls.

These results contribute to understanding of intergenerational influences on childhood psychopathology in several key ways. First, though biological embedding of stress across the life course has a growing evidence base, maternal history of adversity has rarely been considered in prenatal programming studies of child mental health. Simultaneous examination of maternal exposure to stressors during sensitive periods of her own biological and psychosocial development—childhood and pregnancy—allows understanding of unique contributions from mothers’ histories, with current results highlighting the apparent long-term associations of prenatal stress exposure with offspring mental health after accounting for maternal childhood trauma. Second, while there are notable exceptions (e.g., Madigan et al., 2015; Maselko et al., 2016; Ramchandani et al., 2010), much of the empirical work on maternal stress and childhood mental health has used mostly White, middle- to upper-income samples, with many residing in countries with vastly different healthcare and family support systems compared to the United States (e.g., Norway, Canada). Our use of an epidemiological sample of mother-child dyads from six distinct geographic regions of the U.S., reporting a wide range of household income and education levels, with almost half of the women identifying as a person of color, demonstrated replication of PSLE effects in this sample exposed to U.S. culture, policies, and perinatal care. Confidence regarding the robustness of the current findings is bolstered by sensitivity analyses demonstrating the strong consistency of the observed effects when the sample is comprised of various subsets of the seven sites, and when comparing imputed to complete-case dataset results. Lastly, this multi-reporter study addresses a significant limitation of prior research on maternal stress exposure and child mental health related to shared reporter across the predictor and outcome measures (e.g., maternal stress and child anxiety). Due to the difficultly of comprehensive measurement of childhood mental health in epidemiological cohorts because of time constraints on data collection and developmentally appropriate linguistic and cognitive limitations in young children, many studies (including our own, at times) opt for parent-report of child psychological functioning, which may be discrepant from child self-reports (De Los Reyes et al., 2015), as symptoms may be less apparent to caregivers, relative to externalizing behaviors (Kolko & Kazdin, 1993). Thus, our detection of associations using child-report of anxiety and depression symptoms advances confidence in the burgeoning evidence related to intergenerational stress-health associations.

We observed that maternal PSLE was a robust predictor of childhood anxiety and depression, over and above maternal CTE, sociodemographic confounders (e.g., household income, maternal education, race), and factors potentially on the mechanistic pathway between maternal prenatal stress and child mental health (e.g., birth outcomes, maternal depression). In addition, maternal PSLE predicted likelihood of clinically significant child anxiety symptoms. These effects of prenatal stress are consistent with the DOHaD framework, whereby the developing fetus is impacted by maternal experiences and behaviors during pregnancy in a manner that promotes fetal survival but affects long-term health (Barker, 1998; Van den Bergh et al., 2020). Our research group has also demonstrated the dual effects of maternal PSLE and CTE on parent-reported early childhood (age 4–6) total behavior problems in this sample (Bush et al., under review). The effect estimates obtained in the current analyses are notably smaller than those found in early childhood, which indicates a possible attenuation of the effects of maternal childhood trauma and pregnancy stress as their children age, suggests potential for differential impacts of pregnancy stress on internalizing and externalizing problems, and underscores the value of multi-reporter analyses in future work.

Contrary to prior findings (Rowell & Neal-Barnett, 2022), maternal CTE was not a significant predictor of either child anxiety or depression in our primary analytic models. The current study is the first to leverage child self-report of internalizing in this line of inquiry, and the null findings suggest that maternal CTE may not have lasting impacts on child internalizing when children, as opposed to caregivers, are asked to report on their symptoms. There are, however, two important considerations in the interpretation of these associations. First, unlike prior examinations of maternal CTE and offspring internalizing, our primary models analyzed CTE simultaneously with PSLE. In our unadjusted models, when PSLE was not included as a simultaneous predictor of child internalizing, CTE did appear to have a weak bivariate association with depression symptoms. This attenuation of the CTE effect after inclusion of PSLE suggests that covariance between maternal childhood and pregnancy stress exposures (recall they were mildly correlated) may have contributed to attenuated effects. It is also possible that the more proximal timing of PSLE to childhood internalizing, relative to the more distal timing of the CTE, contributed to its more potent effect on child internalizing problems. Second, the 3-item CTE measure assessed only three major types of childhood traumatic exposures (physical and sexual abuse, exposure to violence), and wording on the sexual abuse item did not include exposure to childhood sexual abuse between the ages of 13 and 18. Thus, though these three exposures reflect significant traumatic exposures and their sum score has previously been associated with a variety of maternal-child outcomes in one of the cohorts of this sample (Adgent et al., 2019; Ahmad et al., 2021; Browne et al., 2022; Roubinov et al., 2022; Steine et al., 2020), they may not capture the full range of maternal childhood adverse experiences that contribute to the intergenerational transmission of stress. In addition, the PSLE measure used here included both interpersonal as well as economic adversities, which may provide a more complete picture of stress exposure. Thus, although the current null CTE findings within this strong study design add much-needed evidence to this field of study and point to the potential for maternal childhood trauma having attenuating impacts on offspring mental health over time, future work is needed to evaluate these associations with a more comprehensive measure of maternal childhood stress exposure.

Importantly, our findings in this large sample did not provide evidence for sex differences in the associations between maternal stress exposures and child internalizing problems. Prior investigations of sex differences in vulnerability to maternal stress exposure have yielded inconsistent results; while some have found girls to be at greater risk of internalizing problems (Davis & Sandman, 2012), others have found greater internalizing risk among boys (Letourneau et al., 2019), and yet others have found no significant sex differences (O’Donnell et al., 2014). Some of this inconsistency is likely due to variability in study design across prior studies, including in measurement of maternal stress, child age when internalizing problems were assessed, and sample size. The smaller (Davis & Sandman, 2012) or developmentally earlier (e.g., age 2, Letourneau et al., 2019) studies found support for sex differences, but of note, O’Donnell et al.’s (2014) study of 7,944 children from 4 to 13 years of age did not. In this first study to date to utilize child self-report of internalizing symptoms, in our sample of 1,389 dyads, we found that maternal stress exposures did not differentially associate with boys’ and girls’ self-reported anxiety and depression at age 8. These results increase the weight of evidence supporting a lack of sex-specific effects in middle childhood. Examinations at adolescent timepoints are important to fill in the understanding across developmental course.

Results from exploratory analyses on the potential protective effect of neighborhood resources and opportunities were also null. Our measure of neighborhood quality, the Childhood Opportunity Index (COI), is a multi-sectoral, empirically-derived summary measure comprised of 29 indicators that measure neighborhood-based opportunities for children (e.g., neighborhood/school poverty, quality of early childhood education centers, access to green space); however, the COI’s scoring and item weighting results in a measure that primarily reflects socioeconomic factors in a neighborhood (Noelke et al., 2020). As neighborhood poverty is not distributed equally and is a sequela of de facto residential racial segregation via redlining (Heard-Garris et al., 2021), it is possible that our analyses obscured the main or moderating impact of neighborhood on internalizing problems by covarying for race and ethnicity. In sensitivity analyses excluding the race/ethnicity covariate, the COI interaction remained null, but the main effect of neighborhood quality on child anxiety reached statistical significance, such that higher neighborhood quality was associated with lower levels of anxiety. Given the change in results subsequent to omission of race/ethnicity from models, future work to untangle intersections between structural racism and neighborhood quality will improve understanding of neighborhood effects and their potential to shape maternal stress exposure effects on children’s mental health. In addition, ascertaining neighborhood effects is complex as a number of dimensions, such as conceptual and operational definition and measurement of neighborhood, influence detection. Our objectively-based measure had notable strengths, yet evidence demonstrates that perceptions of neighborhood boundaries and qualities vary greatly by individuals (Lee et al., 2017), and our study did not capture individual family-level exposures to or perception of neighborhood attributes, nor did it assess factors such as community cohesion, which is known to play an important role in healthy child development (Delany-Brumsey et al., 2014). Findings here provide an initial step in the quest for identifying modifiable structural level factors that can buffer the impact of stress across generations, but additional investigation of neighborhood protective factors is needed.

Several limitations should be considered in the interpretation of current findings. First, maternal stress exposure during childhood and pregnancy were both assessed retrospectively. Although prospective collection of stress exposure is ideal, retrospective reports of significant life events (e.g., severe illness, death of a close relative, or relationship changes) have been found to be valid and robust to recall bias over time (Krinsley et al., 2003; Ramos et al., 2020). Second, because our exposure measures captured different multi-domain stressors (3 items of childhood traumatic events vs. 14 items of a range of pregnancy stressful life events), cannot be used to interpret relative effect sizes across maternal exposure developmental windows. Future research with more comprehensive measurement of CTE is needed to directly compare stressor exposure in these two life course periods, as well as assessment of total lifetime stress exposure of mothers from birth through pregnancy, and during the postpartum period, which were not measured in a harmonizable manner across the current cohorts. Such inclusion of total lifetime stressors, in addition to childhood and prenatal stressors, would illuminate the role of timing and sensitive periods for the intergenerational transmission of stress on health. In addition, both of the stress exposure measures assessed only whether the mother had experienced adverse events during the specified time frames (childhood and pregnancy), not how frequent or severe the exposures were, qualities of stress exposure that are also known to impact health (Shields et al., 2022). Third, while the current measures of anxiety and depression have been validated in eight-year-old children, it should be noted that there is considerable variability in emotional knowledge and awareness at this age. A multi-informant approach, as well as clinical interviewing, would enhance confidence in these findings. Fourth, while the current examination conceptualized maternal depression as a mediator of associations between maternal stress exposures and child internalizing symptoms, maternal postnatal depression may also be a key moderator (Eckshtain et al., 2019) and should be examined as such in future research. Last, the estimated effects from our analytic models may be biased by unmeasured confounding variables. The intended use of the current findings is to inform future studies, including prevention and intervention trials, and clinical programs of the importance of intergenerational perspectives in the development of child psychopathology.

Our findings related to maternal exposure to childhood trauma suggest that it may not have enduring impact on child internalizing in analyses adjusted for prenatal stress exposure, sociodemographic factors, and other potential confounders. Although this finding is not conclusive, it is promising in that it suggests that women with childhood adversity histories may not carry mental health risk forward to offspring. On the other hand, our findings do point to maternal exposure to stressful life events during pregnancy as an important target of prevention and intervention efforts. Reduction in the rate of women’s exposure to major life stressors during the pregnancy period must be a public health priority, to improve women’s health but also to break cycles of adversity and endow future generations with foundations of health (Leckman, 2017). That said, many thriving families have a caregiver with a history of adversity who, nonetheless, demonstrates resilience. Policies that have evidence as being beneficial to expectant and new families include enhanced perinatal Medicaid coverage (Roman et al., 2014), increased parental leave (Jou et al., 2018), and perinatal cash transfer programs (Troller-Renfree et al., 2022). In addition, programs that help reduce women’s distress during pregnancy have been shown to have intergenerational benefits (Noroña-Zhou et al., 2022). Current findings suggest that these policies and programs may benefit the prevention childhood internalizing problems. Further inquiry into modifiable, structural-level health-promoting factors that influence intergenerational pathways underlying health is needed inform specific policies to address child mental health problems.

## Electronic Supplementary Material

Below is the link to the electronic supplementary material.

Supplementary Material 1

Supplementary Material 2

## Footnotes

1 In this paper, we use the word “women” throughout, to refer to pregnant people and birthing parents, though we recognize that people who have a uterus but do not identify as cisgender women may carry pregnancies. This decision was based on study design, as “pregnant women” were recruited into the study. More inclusive recruitment is an important future direction of this line of work.

2 Race is a political and social construct that often serves as a proxy for the impact of racist practices and structural inequality, it is not a biological construct (Bryant et al., 2022) and thus is examined in the current paper with this premise in mind.
