# Supplementary material for: A Multi-Cohort Examination of the Independent Contributions of Maternal Childhood Adversity and Pregnancy Stressors to the Prediction of Children’s Anxiety and Depression
Source: Res Child Adolesc Psychopathol. 2022 Dec 3;51(4):497–512. doi: 10.1007/s10802-022-01002-3 (PMC10017630; doi:10.1007/s10802-022-01002-3)
Supplement: Supplementary file 1 — Supplementary Material 1 [file 10802_2022_1002_MOESM1_ESM.docx]

**Supplemental Material for**

A Multi-Cohort Examination of the Independent Contributions of Maternal Childhood Adversity and Pregnancy Stressors to the Prediction of Children’s Anxiety and Depression

**Supplemental Method**

**Pregnancy stressful life events (PSLE) measure.** The PSLE survey was comprised of the following 14 items. Mothers reported whether they had experienced each event during pregnancy.

1. A family member was hospitalized
2. Death of a close friend/family member
3. Moving to a new address
4. Loss of job/employment
5. Partner lost their job
6. Participant/partner had a reduction in work hours or pay
7. Problems paying the rent/mortgage or other bills
8. Separation/divorce from partner
9. Was apart from partner due to military deployment or extended work-related travel
10. Argued with partner more than usual
11. Partner did not want participant to be pregnant
12. Close friend/family member had a problem with drinking/drugs
13. Participant/partner was incarcerated
14. Participant was homeless.

**Regression Model Covariate Details**

A series of three regression models included three additive sets of covariates. Model 1 included methodological variables that might serve as confounders. Model 2 included those and potentially confounding variables at the family-, parent-, and child-level. Models 2a included all covariates from Models 1 and 2, and additional variables potentially on the mechanistic path between maternal stress exposure and child mental health.

Model 1 (minimally-adjusted model): Given the multi-site nature of the total study sample, a variable capturing site was comprised of the following categories: Memphis, TN (CANDLE), San Francisco, CA (TIDES), Minneapolis, MN (TIDES), Rochester, NY (TIDES), Seattle, WA (TIDES), Seattle, WA (GAPPS), Yakima, WA (GAPPS).

Model 2 (fully-adjusted model): Added covariates included a measure of family income, adjusted for household size, region of the country, and inflation. Covaried maternal factors were age, parity (number of previous pregnancies), pre-pregnancy body mass index, level of education (Less than high school, High school diploma or GED, Vocational or Technical school after high school, some College [no degree] or Associate Degree, College Graduate or Baccalaureate degree, Masters Degree, Doctoral-level/professional degree). Covaried child factors were age at outcome, year of birth, biological sex assigned at birth, race/ethnicity^[[1]](#footnote-1)^ (White, Black/African American, Hispanic/Latinx, or other).

Model 2a (fully-adjusted + possible mechanisms model): Additional covariates added in Model 2a were variables potentially on the mechanistic path between maternal stress exposures and child internalizing problems. Potential mechanisms adjusted for in Model 2a were: maternal cigarette smoking during pregnancy (yes/no), child gestational age at birth (days), whether the child was breastfed (yes/no), and maternal self-reported depression at the child age 8 visit. Maternal depression was assessed with the Patient Reported Outcome Measurement Information System (PROMIS) Depression Short Form (PROMIS-D-8) in the TIDES and GAPPS cohorts, which was developed by the National Institutes of Health to measure patient-reported depression symptoms relevant to a range of chronic diseases (Cella et al., 2010; Teresi et al., 2010). This 8-item measure inquires about the frequency of depression symptoms (e.g., sadness, worthlessness, anhedonia) in the previous week. In the CANDLE cohort, maternal depression was assessed with the 6-item depression subscale of the Brief Symptom Inventory (BSI; Derogatis & Melisaratos, 1983), which has been linked to the PROMIS-D-8 via empirical work (Kaat et al., 2017). A crosswalk table has been developed to harmonize these two measures, converting a BSI-depression subscale score to a PROMIS-D-8 t-score. The PROMIS-D-8 has demonstrated good inter-item reliability (range .74-.84) and convergent validity with other established measures of depression (e.g., CES-D and PHQ-9 (Amtmann et al., 2014).

**Supplemental References**

Amtmann, D., Kim, J., Chung, H., Bamer, A. M., Askew, R. L., Wu, S., Cook, K. F., & Johnson, K. L. (2014). Comparing CESD-10, PHQ-9, and PROMIS Depression Instruments in Individuals with Multiple Sclerosis. *Rehabilitation Psychology*, *59*(2), 220–229. https://doi.org/10.1037/a0035919

Bryant, B. E., Jordan, A., & Clark, U. S. (2022). Race as a Social Construct in Psychiatry Research and Practice. *JAMA Psychiatry*, *79*(2), 93–94. https://doi.org/10.1001/jamapsychiatry.2021.2877

Cella, D., Riley, W., Stone, A., Rothrock, N., Reeve, B., Yount, S., Amtmann, D., Bode, R., Buysse, D., Choi, S., Cook, K., DeVellis, R., DeWalt, D., Fries, J. F., Gershon, R., Hahn, E. A., Lai, J.-S., Pilkonis, P., Revicki, D., … Hays, R. (2010). The Patient-Reported Outcomes Measurement Information System (PROMIS) developed and tested its first wave of adult self-reported health outcome item banks: 2005–2008. *Journal of Clinical Epidemiology*, *63*(11), 1179–1194. https://doi.org/10.1016/j.jclinepi.2010.04.011

Derogatis, L. R., & Melisaratos, N. (1983). The Brief Symptom Inventory: An introductory report. *Psychological Medicine*, *13*(3), 595–605.

Kaat, A. J., Newcomb, M. E., Ryan, D. T., & Mustanski, B. (2017). Expanding a common metric for depression reporting: Linking two scales to PROMIS^®^ depression. *Quality of Life Research*, *26*(5), 1119–1128. https://doi.org/10.1007/s11136-016-1450-z

Teresi, J. A., Ocepek-Welikson, K., Kleinman, M., Eimicke, J. P., Crane, K., Jones, R. N., Lai, J., Choi, S. W., Hays, R. D., Reise, S. P., Pilkonis, P. A., & Cella, D. (2010). *Analysis of Differential Item Functioning in the Depression Item Bank from the Patient Reported Outcome Measurement Information System (PROMIS): An Item Response Theory Approach*. 35.

**Supplemental Tables**

**Contents:**

1. Table S1: Descriptives of outcome measures
2. Table S2: Descriptives of exposure measures
3. Table S3: Anxiety symptoms regression results: primary and extended covariate models, all coefficients
4. Table S4: Depression symptoms regression results: primary and extended covariate models, all coefficients
5. Table S5: Logistic regressions predicting clinically significant levels of child anxiety and depression symptoms
6. Figure S1: Visual summary of primary anxiety model results and sensitivity analyses
7. Figure S2: Visual summary of primary depression model results and sensitivity analyses

1. Race is a political and social construct that often serves as a proxy for the impact of racist practices and structural inequality, it is not a biological construct (Bryant et al., 2022) and thus is examined in the current paper with this premise in mind. [↑](#footnote-ref-1)
