# Supplementary material for: A Multi-Cohort Examination of the Independent Contributions of Maternal Childhood Adversity and Pregnancy Stressors to the Prediction of Children’s Anxiety and Depression
Source: Res Child Adolesc Psychopathol. 2022 Dec 3;51(4):497–512. doi: 10.1007/s10802-022-01002-3 (PMC10017630; doi:10.1007/s10802-022-01002-3)
Supplement: Supplementary file 2 — Supplementary Material 2 [file 10802_2022_1002_MOESM2_ESM.docx]

Supplemental Table S1. Descriptives of child self-reported anxiety and depression symptoms

| Child Age 8 Internalizing Symptoms | Overall, N = 1,389^1^ | CANDLE, N = 824 | GAPPS, N = 119 | TIDES, N = 446 | p-value^2^ |
| --- | --- | --- | --- | --- | --- |
| **CDI-2 Depression raw score** |  |  |  |  | <0.001 |
| N | 1,365 | 821 | 116 | 428 |  |
| Mean (SD) | 9 (7) | 9 (6) | 6 (4) | 8 (7) |  |
| Median [IQR] | 7 [4, 12] | 8 [4, 13] | 5 [3, 9] | 7 [3, 11] |  |
| Range | 0, 41 | 0, 41 | 0, 17 | 0, 38 |  |
| Missing | 24 | 3 | 3 | 18 |  |
| **CDI-2 Depression clinical threshold, n (%)** | 200(14.7) | 136 (16.1) | 7 (6.1) | 57 (13.3) | 0.005 |
| Missing | 24 | 3 | 3 | 18 |  |
| **SCARED Anxiety total score** |  |  |  |  | <0.001 |
| N | 1,365 | 822 | 113 | 430 |  |
| Mean (SD) | 26 (14) | 28 (14) | 21 (11) | 22 (14) |  |
| Median [IQR] | 24 [15, 35] | 26 [17, 38] | 19 [12, 29] | 20 [11, 30] |  |
| Range | 0, 82 | 0, 80 | 0, 56 | 0, 82 |  |
| Missing | 24 | 2 | 6 | 16 |  |
| **SCARED Anxiety score >=30, n (%)** | 507 (37) | 363 (44) | 27 (24) | 117 (27) | <0.001 |
| Missing | 24 | 2 | 6 | 16 |  |
| ^1^c("N", "Mean (SD)", "Median [IQR]", "Range"); n (%) | | | | | |
| ^2^Kruskal-Wallis rank sum test; Pearson's Chi-squared test | | | | | |

Supplemental Table S2. Descriptives of maternal self-reported stress exposures

| Exposure | Overall, N = 1,389 | CANDLE, N = 824 | GAPPS, N = 119 | TIDES, N = 446 | p-value^1^ |
| --- | --- | --- | --- | --- | --- |
| **CTE (0-3)** |  |  |  |  | 0.030 |
| N | 1,315 | 774 | 119 | 422 |  |
| Mean (SD) | 0.51 (0.79) | 0.52 (0.78) | 0.38 (0.78) | 0.52 (0.82) |  |
| Median [IQR] | 0.00 [0.00, 1.00] | 0.00 [0.00, 1.00] | 0.00 [0.00, 0.00] | 0.00 [0.00, 1.00] |  |
| Range | 0.00, 3.00 | 0.00, 3.00 | 0.00, 3.00 | 0.00, 3.00 |  |
| Missing | 74 | 50 | 0 | 24 |  |
| **SLE (0-14)** |  |  |  |  | 0.001 |
| N | 1,350 | 818 | 115 | 417 |  |
| Mean (SD) | 1.55 (1.81) | 1.70 (1.88) | 1.37 (1.90) | 1.30 (1.59) |  |
| Median [IQR] | 1.00 [0.00, 2.00] | 1.00 [0.00, 3.00] | 1.00 [0.00, 2.00] | 1.00 [0.00, 2.00] |  |
| Range | 0.00, 10.00 | 0.00, 10.00 | 0.00, 9.00 | 0.00, 8.00 |  |
| Missing | 39 | 6 | 4 | 29 |  |
| ^1^Kruskal-Wallis rank sum test | | | | | |

*Notes*. CTE = childhood traumatic events; PSLE = pregnancy stressful life events

Table S3. Pearson correlations among key study variables

| Variable | CTE | PSLE | COI | Anxiety | Depression | Sex |
| --- | --- | --- | --- | --- | --- | --- |
| CTE |  |  |  |  |  |  |
| SLE | .26* |  |  |  |  |  |
| COI | -.18* | -.22* |  |  |  |  |
| Anxiety | .06 | .15* | -.28* |  |  |  |
| Depression | .08* | .13* | -.22* | .53* |  |  |
| Sex  (girl = 0, boy = 1) | .00 | -.03 | .02 | -.06* | .04 |  |

*Notes*. CTE = childhood traumatic events; PSLE = pregnancy stressful life events, * = p<.05

Table S4. Anxiety symptoms regression results: primary and extended covariate models, all coefficients

|  | Minimally-Adjusted | Primary Model | Effect Modification | Extended Models | |
| --- | --- | --- | --- | --- | --- |
|  | Model 1 | Model 2 | Model 3 | Model 2a | Model 2b |
| CTE | 0.01 | -0.02 | -0.03 | -0.02 | -0.02 |
|  | (-0.05, 0.06) | (-0.08, 0.04) | (-0.11, 0.05) | (-0.08, 0.03) | (-0.07, 0.04) |
| PSLE | 0.12 *** | 0.09 ** | 0.13 ** | 0.08 ** | 0.08 ** |
|  | (0.06, 0.17) | (0.03, 0.14) | (0.05, 0.21) | (0.02, 0.14) | (0.03, 0.14) |
| Child age at outcome | -0.04 | -0.09 * | -0.09 * | -0.09 * | -0.09 * |
|  | (-0.09, 0.01) | (-0.17, -0.01) | (-0.17, -0.01) | (-0.17, -0.01) | (-0.17, -0.01) |
| Sex (girl = 0, boy = 1) | -0.11 * | -0.13 * | -0.13 * | -0.13 * | -0.13 * |
|  | (-0.21, -0.01) | (-0.23, -0.03) | (-0.23, -0.03) | (-0.23, -0.02) | (-0.23, -0.03) |
| CTE x Sex |  |  | 0.02 |  |  |
|  |  |  | (-0.09, 0.13) |  |  |
| PSLE x Sex |  |  | -0.09 |  |  |
|  |  |  | (-0.20, 0.02) |  |  |
| Child race and ethnicity: Non-Hispanic, white |  | -0.25 ** | -0.25 ** | -0.24 ** |  |
|  |  | (-0.42, -0.08) | (-0.42, -0.08) | (-0.42, -0.07) |  |
| Child race and ethnicity: Non-Hispanic, more than one race |  | -0.03 | -0.03 | -0.03 |  |
|  |  | (-0.25, 0.19) | (-0.25, 0.18) | (-0.24, 0.19) |  |
| Child race and ethnicity: Hispanic |  | 0.11 | 0.11 | 0.11 |  |
|  |  | (-0.13, 0.35) | (-0.13, 0.34) | (-0.13, 0.35) |  |
| Maternal age |  | -0.05 | -0.04 | -0.05 | -0.05 |
|  |  | (-0.12, 0.03) | (-0.12, 0.03) | (-0.12, 0.03) | (-0.13, 0.02) |
| Maternal education: High school/GED |  | 0.04 | 0.05 | 0.04 | 0.05 |
|  |  | (-0.25, 0.34) | (-0.25, 0.34) | (-0.26, 0.34) | (-0.25, 0.35) |
| Maternal education: Associates/technical school |  | 0.05 | 0.06 | 0.04 | 0.06 |
|  |  | (-0.26, 0.37) | (-0.26, 0.37) | (-0.28, 0.36) | (-0.26, 0.38) |
| Maternal education: College degree |  | -0.01 | -0.01 | -0.02 | -0.01 |
|  |  | (-0.33, 0.32) | (-0.33, 0.31) | (-0.34, 0.31) | (-0.34, 0.31) |
| Maternal education: Graduate or professional degree |  | -0.05 | -0.05 | -0.06 | -0.05 |
|  |  | (-0.39, 0.29) | (-0.39, 0.29) | (-0.40, 0.29) | (-0.39, 0.28) |
| Pre-pregnancy BMI |  | -0.01 | -0.00 | -0.01 | -0.00 |
|  |  | (-0.06, 0.05) | (-0.06, 0.05) | (-0.06, 0.05) | (-0.06, 0.05) |
| Birth order (0 - 13) |  | 0.02 | 0.01 | 0.01 | 0.02 |
|  |  | (-0.02, 0.05) | (-0.02, 0.05) | (-0.02, 0.05) | (-0.02, 0.06) |
| Household count: 3 |  | 0.27 | 0.27 | 0.28 | 0.26 |
|  |  | (-0.06, 0.60) | (-0.05, 0.60) | (-0.06, 0.61) | (-0.07, 0.59) |
| Household count: 4 |  | 0.38 * | 0.38 * | 0.39 * | 0.35 * |
|  |  | (0.06, 0.69) | (0.06, 0.69) | (0.07, 0.71) | (0.03, 0.66) |
| Household count: 5 |  | 0.41 * | 0.41 * | 0.42 * | 0.38 * |
|  |  | (0.08, 0.73) | (0.09, 0.74) | (0.09, 0.74) | (0.05, 0.71) |
| Household count: 6+ |  | 0.27 | 0.28 | 0.28 | 0.24 |
|  |  | (-0.07, 0.62) | (-0.06, 0.63) | (-0.06, 0.63) | (-0.11, 0.59) |
| log(Household income) |  | -0.11 | -0.11 | -0.11 | -0.12 |
|  |  | (-0.51, 0.30) | (-0.51, 0.29) | (-0.51, 0.29) | (-0.53, 0.28) |
| log(Income) x Household size=3 |  | 0.05 | 0.05 | 0.06 | 0.06 |
|  |  | (-0.36, 0.47) | (-0.36, 0.47) | (-0.35, 0.47) | (-0.36, 0.49) |
| log(Income) x Household size=4 |  | -0.03 | -0.02 | -0.02 | -0.03 |
|  |  | (-0.43, 0.38) | (-0.43, 0.38) | (-0.43, 0.38) | (-0.44, 0.38) |
| log(Income) x Household size=5 |  | 0.07 | 0.06 | 0.07 | 0.07 |
|  |  | (-0.35, 0.48) | (-0.35, 0.48) | (-0.34, 0.48) | (-0.35, 0.49) |
| log(Income) x Household size=6+ |  | -0.05 | -0.05 | -0.05 | -0.05 |
|  |  | (-0.45, 0.36) | (-0.46, 0.36) | (-0.45, 0.36) | (-0.46, 0.36) |
| COI |  | -0.03 | -0.02 | -0.03 | -0.09 * |
|  |  | (-0.12, 0.06) | (-0.12, 0.07) | (-0.13, 0.06) | (-0.17, -0.00) |
| Maternal depression |  |  |  | 0.03 |  |
|  |  |  |  | (-0.03, 0.09) |  |
| Pregnancy smoking (any/none) |  |  |  | -0.05 |  |
|  |  |  |  | (-0.24, 0.13) |  |
| Gestational age (days) |  |  |  | -0.04 |  |
|  |  |  |  | (-0.12, 0.04) |  |
| Birthweight (grams) |  |  |  | -0.00 |  |
|  |  |  |  | (-0.08, 0.07) |  |
| Breastfeeding (any/none) |  |  |  | 0.03 |  |
|  |  |  |  | (-0.11, 0.17) |  |
| Cohort birth year: 2008 |  | -0.13 | -0.14 | -0.13 | -0.15 |
|  |  | (-0.46, 0.19) | (-0.46, 0.18) | (-0.45, 0.19) | (-0.48, 0.17) |
| Cohort birth year: 2009 |  | -0.08 | -0.08 | -0.09 | -0.08 |
|  |  | (-0.41, 0.25) | (-0.41, 0.25) | (-0.42, 0.25) | (-0.42, 0.25) |
| Cohort birth year: 2010 |  | -0.12 | -0.12 | -0.12 | -0.12 |
|  |  | (-0.49, 0.25) | (-0.49, 0.25) | (-0.50, 0.25) | (-0.49, 0.26) |
| Cohort birth year: 2011 |  | -0.11 | -0.12 | -0.12 | -0.11 |
|  |  | (-0.49, 0.27) | (-0.50, 0.27) | (-0.50, 0.27) | (-0.50, 0.27) |
| Cohort birth year: 2012 |  | -0.20 | -0.21 | -0.19 | -0.19 |
|  |  | (-0.66, 0.26) | (-0.67, 0.25) | (-0.66, 0.27) | (-0.66, 0.27) |
| Cohort birth year: 2013 |  | -0.39 | -0.40 | -0.36 | -0.40 |
|  |  | (-0.93, 0.14) | (-0.94, 0.14) | (-0.90, 0.18) | (-0.94, 0.14) |
| Site: Memphis, TN | -0.58 *** | -0.15 | -0.14 | -0.18 | -0.12 |
|  | (-0.76, -0.39) | (-0.44, 0.14) | (-0.44, 0.15) | (-0.48, 0.12) | (-0.42, 0.17) |
| Site: San Francisco, CA | -0.57 *** | -0.15 | -0.15 | -0.18 | -0.19 |
|  | (-0.75, -0.38) | (-0.44, 0.13) | (-0.43, 0.13) | (-0.47, 0.11) | (-0.47, 0.09) |
| Site: Minneapolis, MN | -0.03 | 0.12 | 0.13 | 0.10 | 0.10 |
|  | (-0.22, 0.16) | (-0.16, 0.40) | (-0.15, 0.41) | (-0.18, 0.38) | (-0.18, 0.37) |
| Site: Rochester, NY | -0.51 *** | -0.20 | -0.19 | -0.22 | -0.19 |
|  | (-0.72, -0.30) | (-0.48, 0.09) | (-0.48, 0.09) | (-0.50, 0.07) | (-0.47, 0.09) |
| Site: Seattle, WA (TIDES) | -0.45 *** | -0.04 | -0.03 | -0.14 | -0.05 |
|  | (-0.67, -0.23) | (-0.35, 0.27) | (-0.34, 0.28) | (-0.47, 0.19) | (-0.36, 0.26) |
| Site: Seattle, WA (GAPPS) | -0.72 *** | -0.40 + | -0.39 + | -0.41 + | -0.45 * |
|  | (-1.07, -0.37) | (-0.81, 0.02) | (-0.81, 0.02) | (-0.83, 0.01) | (-0.86, -0.03) |
| R^2^ | 0.09 | 0.15 | 0.15 | 0.15 | 0.14 |

*Notes.* CTE = childhood traumatic events; PSLE = pregnancy stressful life events; COI = Childhood Opportunity Index

Table S5. Depression symptoms regression results: primary and extended covariate models, all coefficients

|  | Minimally-Adjusted | Primary Model | Effect Modification | Extended Models |  |
| --- | --- | --- | --- | --- | --- |
|  | Model 1 | Model 2 | Model 3 | Model 2a | Model 2b |
| CTE | 0.02 | -0.01 | 0.02 | -0.02 | -0.01 |
|  | (-0.03, 0.08) | (-0.06, 0.05) | (-0.05, 0.10) | (-0.08, 0.04) | (-0.06, 0.05) |
| PSLE | 0.11 *** | 0.08 ** | 0.12 ** | 0.06 * | 0.08 ** |
|  | (0.06, 0.17) | (0.03, 0.14) | (0.05, 0.20) | (0.00, 0.12) | (0.03, 0.14) |
| Child age at outcome | -0.07 * | -0.08 + | -0.08 + | -0.09 * | -0.08 + |
|  | (-0.12, -0.01) | (-0.16, 0.00) | (-0.16, 0.00) | (-0.17, -0.01) | (-0.16, 0.00) |
| Sex (girl = 0, boy = 1) | 0.10 + | 0.09 + | 0.09 + | 0.09 + | 0.09 + |
|  | (-0.00, 0.20) | (-0.01, 0.19) | (-0.02, 0.19) | (-0.01, 0.19) | (-0.02, 0.19) |
| CTE x Sex |  |  | -0.06 |  |  |
|  |  |  | (-0.17, 0.04) |  |  |
| PSLE x Sex |  |  | -0.08 |  |  |
|  |  |  | (-0.19, 0.02) |  |  |
| Child race and ethnicity: Non-Hispanic, white |  | -0.09 | -0.09 | -0.11 |  |
|  |  | (-0.26, 0.08) | (-0.26, 0.08) | (-0.28, 0.06) |  |
| Child race and ethnicity: Non-Hispanic, more than one race |  | 0.05 | 0.05 | 0.03 |  |
|  |  | (-0.17, 0.27) | (-0.17, 0.27) | (-0.19, 0.25) |  |
| Child race and ethnicity: Hispanic |  | 0.09 | 0.09 | 0.07 |  |
|  |  | (-0.15, 0.34) | (-0.16, 0.33) | (-0.18, 0.31) |  |
| Maternal age |  | -0.06 | -0.06 | -0.05 | -0.06 |
|  |  | (-0.13, 0.02) | (-0.13, 0.02) | (-0.13, 0.02) | (-0.13, 0.02) |
| Maternal education: High school/GED |  | 0.17 | 0.18 | 0.19 | 0.17 |
|  |  | (-0.11, 0.46) | (-0.11, 0.47) | (-0.10, 0.48) | (-0.12, 0.45) |
| Maternal education: Associates/technical school |  | 0.12 | 0.13 | 0.13 | 0.12 |
|  |  | (-0.18, 0.43) | (-0.17, 0.43) | (-0.17, 0.44) | (-0.18, 0.42) |
| Maternal education: College degree |  | 0.11 | 0.12 | 0.13 | 0.10 |
|  |  | (-0.20, 0.42) | (-0.19, 0.43) | (-0.19, 0.44) | (-0.21, 0.41) |
| Maternal education: Graduate or professional degree |  | 0.11 | 0.12 | 0.13 | 0.11 |
|  |  | (-0.22, 0.44) | (-0.21, 0.45) | (-0.19, 0.46) | (-0.22, 0.43) |
| Pre-pregnancy BMI |  | 0.07 * | 0.07 ** | 0.07 ** | 0.07 * |
|  |  | (0.01, 0.12) | (0.02, 0.13) | (0.02, 0.13) | (0.01, 0.12) |
| Birth order (0 - 13) |  | 0.02 | 0.02 | 0.02 | 0.02 |
|  |  | (-0.02, 0.06) | (-0.02, 0.06) | (-0.02, 0.06) | (-0.02, 0.06) |
| Household count: 3 |  | 0.17 | 0.18 | 0.16 | 0.17 |
|  |  | (-0.16, 0.50) | (-0.15, 0.51) | (-0.17, 0.50) | (-0.17, 0.50) |
| Household count: 4 |  | 0.33 * | 0.34 * | 0.33 * | 0.32 + |
|  |  | (0.01, 0.64) | (0.02, 0.65) | (0.01, 0.64) | (-0.00, 0.63) |
| Household count: 5 |  | 0.25 | 0.27 | 0.23 | 0.24 |
|  |  | (-0.08, 0.59) | (-0.07, 0.60) | (-0.10, 0.57) | (-0.09, 0.57) |
| Household count: 6+ |  | 0.27 | 0.28 | 0.25 | 0.26 |
|  |  | (-0.08, 0.62) | (-0.07, 0.63) | (-0.10, 0.60) | (-0.09, 0.60) |
| log(Household income) |  | -0.27 | -0.27 | -0.25 | -0.28 |
|  |  | (-0.65, 0.10) | (-0.65, 0.11) | (-0.63, 0.12) | (-0.65, 0.10) |
| log(Income) x Household size=3 |  | 0.17 | 0.16 | 0.17 | 0.17 |
|  |  | (-0.22, 0.56) | (-0.23, 0.56) | (-0.23, 0.56) | (-0.22, 0.57) |
| log(Income) x Household size=4 |  | 0.14 | 0.14 | 0.12 | 0.13 |
|  |  | (-0.25, 0.53) | (-0.26, 0.53) | (-0.27, 0.52) | (-0.26, 0.52) |
| log(Income) x Household size=5 |  | 0.24 | 0.23 | 0.24 | 0.24 |
|  |  | (-0.14, 0.62) | (-0.15, 0.62) | (-0.14, 0.62) | (-0.14, 0.62) |
| log(Income) x Household size=6+ |  | 0.10 | 0.09 | 0.10 | 0.10 |
|  |  | (-0.30, 0.50) | (-0.30, 0.49) | (-0.30, 0.49) | (-0.30, 0.50) |
| COI |  | -0.04 | -0.04 | -0.04 | -0.06 |
|  |  | (-0.13, 0.05) | (-0.13, 0.05) | (-0.13, 0.05) | (-0.15, 0.02) |
| Maternal depression |  |  |  | 0.07 * |  |
|  |  |  |  | (0.01, 0.13) |  |
| Pregnancy smoking (any/none) |  |  |  | 0.19 * |  |
|  |  |  |  | (0.00, 0.38) |  |
| Gestational age (days) |  |  |  | -0.00 |  |
|  |  |  |  | (-0.08, 0.08) |  |
| Birthweight (grams) |  |  |  | -0.04 |  |
|  |  |  |  | (-0.12, 0.03) |  |
| Breastfeeding (any/none) |  |  |  | 0.07 |  |
|  |  |  |  | (-0.07, 0.21) |  |
| Cohort birth year: 2008 |  | -0.01 | -0.00 | 0.01 | -0.02 |
|  |  | (-0.33, 0.32) | (-0.33, 0.32) | (-0.32, 0.33) | (-0.34, 0.31) |
| Cohort birth year: 2009 |  | 0.20 | 0.21 | 0.20 | 0.19 |
|  |  | (-0.14, 0.53) | (-0.13, 0.54) | (-0.13, 0.53) | (-0.14, 0.52) |
| Cohort birth year: 2010 |  | 0.23 | 0.24 | 0.22 | 0.22 |
|  |  | (-0.15, 0.60) | (-0.13, 0.62) | (-0.15, 0.60) | (-0.15, 0.60) |
| Cohort birth year: 2011 |  | 0.14 | 0.14 | 0.12 | 0.13 |
|  |  | (-0.25, 0.52) | (-0.24, 0.53) | (-0.27, 0.50) | (-0.25, 0.52) |
| Cohort birth year: 2012 |  | 0.01 | 0.01 | -0.00 | 0.01 |
|  |  | (-0.46, 0.48) | (-0.46, 0.48) | (-0.47, 0.47) | (-0.46, 0.48) |
| Cohort birth year: 2013 |  | -0.19 | -0.19 | -0.19 | -0.20 |
|  |  | (-0.74, 0.35) | (-0.73, 0.36) | (-0.74, 0.35) | (-0.74, 0.35) |
| Site: Memphis, TN | -0.38 *** | 0.08 | 0.10 | 0.04 | 0.11 |
|  | (-0.57, -0.19) | (-0.22, 0.38) | (-0.20, 0.40) | (-0.27, 0.34) | (-0.19, 0.41) |
| Site: San Francisco, CA | -0.45 *** | -0.04 | -0.03 | -0.07 | -0.05 |
|  | (-0.63, -0.26) | (-0.33, 0.25) | (-0.32, 0.25) | (-0.36, 0.22) | (-0.34, 0.23) |
| Site: Minneapolis, MN | 0.40 *** | 0.56 *** | 0.58 *** | 0.53 *** | 0.56 *** |
|  | (0.21, 0.59) | (0.28, 0.85) | (0.29, 0.86) | (0.24, 0.81) | (0.28, 0.84) |
| Site: Rochester, NY | -0.13 | 0.21 | 0.22 | 0.18 | 0.22 |
|  | (-0.34, 0.08) | (-0.08, 0.50) | (-0.07, 0.51) | (-0.11, 0.47) | (-0.06, 0.51) |
| Site: Seattle, WA (TIDES) | -0.38 *** | 0.07 | 0.08 | -0.03 | 0.07 |
|  | (-0.60, -0.16) | (-0.25, 0.39) | (-0.24, 0.40) | (-0.37, 0.30) | (-0.24, 0.39) |
| Site: Seattle, WA (GAPPS) | -0.59 *** | -0.28 | -0.27 | -0.25 | -0.29 |
|  | (-0.92, -0.25) | (-0.69, 0.13) | (-0.68, 0.14) | (-0.66, 0.16) | (-0.70, 0.12) |
| R^2^ | 0.08 | 0.13 | 0.13 | 0.14 | 0.12 |

*Notes*. CTE = childhood traumatic events; PSLE = pregnancy stressful life events; COI = Childhood Opportunity Index

Table S5. Logistic regressions predicting clinically significant levels of child anxiety and depression symptoms

|  | Anxiety  (SCARED > 30) | | | Depression  (CDI2 > Elevated) | | |
| --- | --- | --- | --- | --- | --- | --- |
| Variable | OR | 95% CI | p-value | OR | 95% CI | p-value |
| CTE (sum 0-3) | 0.96 | 0.82, 1.13 | 0.6 | 1.03 | 0.84, 1.27 | 0.8 |
| PSLE (sum 0-14) | 1.09 | 1.02, 1.17 | 0.012 | 1.07 | 0.98, 1.17 | 0.11 |

*Notes*. CTE = childhood traumatic events; PSLE = pregnancy stressful life events

Supplemental Figure S1. Visual summary of primary anxiety model results and sensitivity analyses


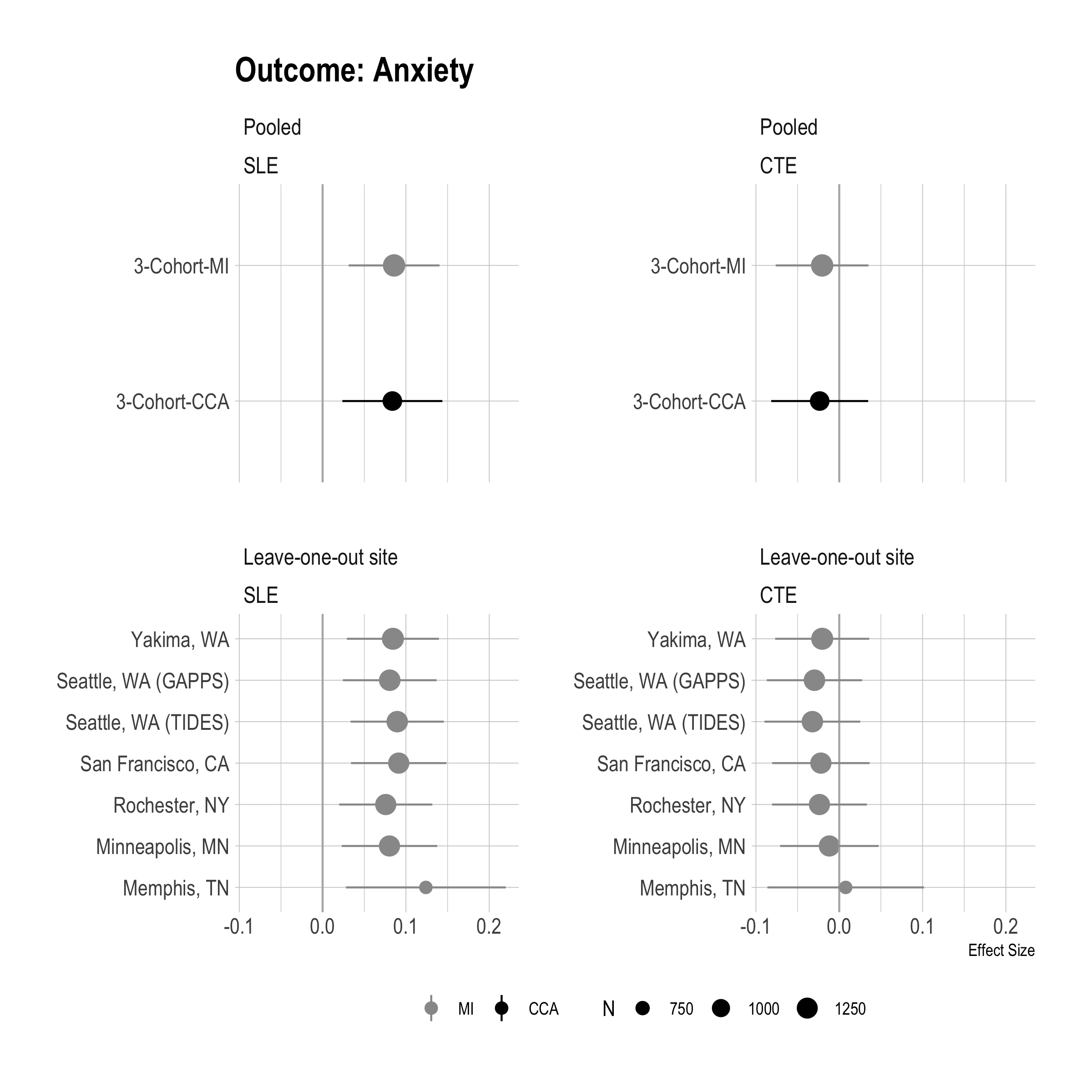


*Notes*. CTE = childhood traumatic events; PSLE = pregnancy stressful life events. Standardized *β* and 95% CI displayed in figures. Estimate for pooled sample using multiply-imputed (MI) data is from primary analysis model 3. Comparison estimates from complete-cases (CCA) and leave-one-out models use same covariate strategy as primary model 3. Plotted point size is area-scaled to each analytic subsample size.

Supplemental Figure S2. Visual summary of primary depression model results and sensitivity analyses


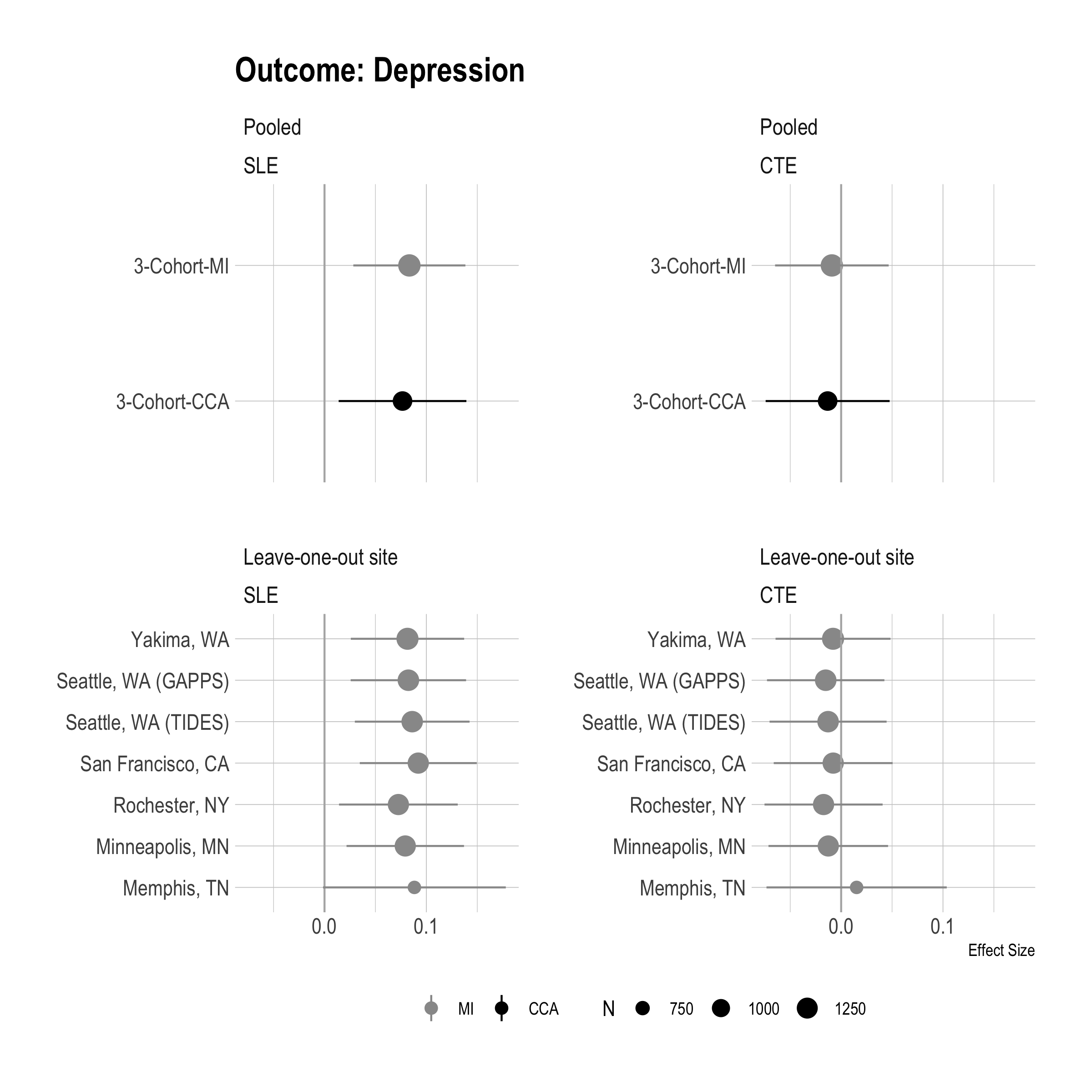


*Notes*. CTE = childhood traumatic events; PSLE = pregnancy stressful life events. Standardized *β* and 95% CI displayed in figures. Estimate for pooled sample using multiply-imputed (MI) data is from primary analysis model 3. Comparison estimates from complete-cases (CCA) and leave-one-out models use same covariate strategy as primary model 3. Plotted point size is area-scaled to each analytic subsample size.
